# Toxicity, Physiological, and Ultrastructural Effects of Arsenic and Cadmium on the Extremophilic Microalga *Chlamydomonas acidophila*

**DOI:** 10.3390/ijerph17051650

**Published:** 2020-03-03

**Authors:** Silvia Díaz, Patricia De Francisco, Sanna Olsson, Ángeles Aguilera, Elena González-Toril, Ana Martín-González

**Affiliations:** 1 Department of Genetics, Physiology and Microbiology, Faculty of Biology, Universidad Complutense de Madrid (UCM), C/José Antonio Novais, 12, 28040 Madrid, Spain; silviadi@bio.ucm.es (S.D.); anamarti@bio.ucm.es (A.M.-G.); 2 Astrobiology Center (INTA-CSIC), Carretera de Ajalvir km 4, Torrejón de Ardoz, 28850 Madrid, Spain; gonzalezte@cab.inta-csic.es; 3 Department of Forest Ecology and Genetics, INIA Forest Research Center (INIA-CIFOR), Carretera de A Coruña km 7.5, 28040 Madrid, Spain; sanna.olsson@helsinki.fi

**Keywords:** arsenic, acidophiles, cadmium, cytotoxicity, extremophiles, ultrastructure, ROS

## Abstract

The cytotoxicity of cadmium (Cd), arsenate (As(V)), and arsenite (As(III)) on a strain of *Chlamydomonas acidophila*, isolated from the Rio Tinto, an acidic environment containing high metal(l)oid concentrations, was analyzed. We used a broad array of methods to produce complementary information: cell viability and reactive oxygen species (ROS) generation measures, ultrastructural observations, transmission electron microscopy energy dispersive x-ray microanalysis (TEM–XEDS), and gene expression. This acidophilic microorganism was affected differently by the tested metal/metalloid: It showed high resistance to arsenic while Cd was the most toxic heavy metal, showing an LC_50_ = 1.94 µM. Arsenite was almost four-fold more toxic (LC_50_= 10.91 mM) than arsenate (LC_50_ = 41.63 mM). Assessment of ROS generation indicated that both arsenic oxidation states generate superoxide anions. Ultrastructural analysis of exposed cells revealed that stigma, chloroplast, nucleus, and mitochondria were the main toxicity targets. Intense vacuolization and accumulation of energy reserves (starch deposits and lipid droplets) were observed after treatments. Electron-dense intracellular nanoparticle-like formation appeared in two cellular locations: inside cytoplasmic vacuoles and entrapped into the capsule, around each cell. The chemical nature (Cd or As) of these intracellular deposits was confirmed by TEM–XEDS. Additionally, they also contained an unexpected high content in phosphorous, which might support an essential role of poly-phosphates in metal resistance.

## 1. Introduction

Freshwater ecosystems are one of the environments most seriously affected by pollution, becoming a major global threat [1,2]. Among the chemical pollutants, heavy metals, mainly produced by industrial activity, are a primary source of contamination. They are a major cause of concern for aquatic environments due to their toxic, persistent, and accumulative properties in organisms, causing devastating effects on the ecological balance of the aquatic environment, and the diversity of aquatic animals [3]. As the metal levels increase, the concern of metal bioaccumulation through the food chain and related human health hazards rises [4]. Since phytoplankton species are the basis of aquatic food webs, there is a considerable body of references on the effects of heavy metals on the growth and development of phytoplankton [5,6,7]. However, most of these studies are related to circumneutral pH water environments polluted by industrial and domestic wastes, in spite of the fact that pH has a considerable effect on the availability and, as a consequence, the toxicity of heavy metals [8].

Indeed, many freshwater bodies worldwide are highly acidic (pH < 3), either due to natural causes or anthropogenic activities such as mining [9,10]. Acid drainages are due mainly to pyrite oxidation, producing significant acidity in lakes and ponds situated in areas impacted by mining, as well as in rivers receiving mine water discharge [11,12]. Additionally, extreme acidic environments tend to contain unusually high concentrations of heavy metals because their solubility increases markedly as the pH decreases [11]. Despite these extreme environmental conditions, a number of prokaryotic and eukaryotic organisms living in these extreme environments have been identified [13]. Microalgae are the dominant eukaryotes found in these ecosystems, green microalgae such as *Chlamydomonas*, *Dunaliella,* and *Chlorella* being the main primary producers [13].

Although acidophilic algae are able to grow at high heavy metal concentrations and low pH, both of which are lethal to most eukaryotes [14], the adaptive mechanisms by which these microorganisms can survive under such environmental conditions are still poorly understood. Proteomic analysis has pointed out the importance of metal and acidity tolerance proteins, such as molecular chaperones of the heat shock protein family [15,16]. Likewise, genomic studies on the acidophiles *Chlamydomonas eustigma* [17] and *Galdieria sulphuraria* [18] have shown that higher expression of heat shock proteins and H^+^-ATPase, the loss of some metabolic pathways that acidify cytosol, and acquisition of metal-detoxifying genes by horizontal gene transfer have played important roles in the adaptation to acidic environments. Furthermore, novel phytochelatin synthase genes from *Dunaliella acidophila* and *Chlamydomonas acidophila* that appear to have a bacterial origin and a response to Cd and Cu, have been identified by transcriptomic approaches [19], although further experimental analyses are needed in order to fully understand their heavy metal resistance mechanisms

To gather information about these issues, in the present study, we investigate the toxic effects of Cd and As on one strain of *Chlamydomonas acidophila* isolated from Río Tinto (Southwest, Spain), one of the most unique examples of extreme acidic environments due to its peculiar microbial ecology, in which eukaryotic organisms are the principal contributors of biomass to the river [20]. Rio Tinto flows through the Iberian Pyrite Belt (Southwest, Spain). It is a natural acidic extreme habitat (pH range between 0.7 and 4.7, mean 2.3), buffered by ferric irons and with high concentrations of dissolved heavy metals and metalloids [20]. These extreme conditions are the result of the metabolic activity of chemolithotrophic prokaryotes living in its waters. The high concentrations of iron- and sulfur-oxidizing bacteria produce the solubilization of sulfidic minerals (mainly FeS_2_), which cause a drastic increment of ferric iron, sulfate and protons concentrations in the water column. Strong acidity has also produced the liberation of many metallic/metalloid cations from metal sulfides [21]. Due to its extreme environmental conditions (e.g., acid pH, high levels of heavy metals/metalloids), it might be expected that poor microbial communities would have been found in Rio Tinto. However, experimental results revealed a high diversity of both prokaryotic [21] and, especially, eukaryotic microorganisms [20].

Metal toxicity depends mainly on the chemical nature, oxidation state, concentration, and bioavailability. In the environment, biological interactions with metal ions become more complex because many external factors (e.g., pH, phosphate, sulphate, geology, organic matter content, redox state, other metals) modulate the available concentration of each metallic species and, therefore, their biotoxicity in a given microhabitat. Cadmium (Cd) is a non-essential heavy metal that induces oxidative stress in many organisms despite its inability to generate free radicals directly [22]. This metal interferes with the structure and function of several molecules with marked endpoints such as cell death and uncontrolled cell proliferation. It has also been proven to be involved in genotoxicity and carcinogenesis [23]. Cd tolerance in microalgae may be influenced by environmental factors; two of them being phosphate and sulfate concentrations. Arsenic (As) is a highly toxic metalloid, widely distributed in soils and aquatic ecosystems, that has been considered as a priority pollutant in many countries [24,25]. Inorganic arsenic tends to be far more toxic than organic arsenic [26]. The major inorganic forms include the trivalent arsenite (As (III)) and the pentavalent arsenate (As (V)) [24]. In microalgae, the uptake of arsenate/arsenite is affected by phosphate concentration in the medium in species-specific ways. As a phosphate analog, arsenate competes with phosphate entering the cells via phosphate transporters. However, other arsenic transport pathways have been discovered as well [27,28].

In this study, the cytotoxicity of cadmium (Cd), arsenate (As(V)) and arsenite (As(III)) on a strain of *C. acidophila*, isolated from Río Tinto, is analyzed by using different methodological approaches: cell viability and ROS generation measures, ultrastructural observations, transmission electron microscopy energy dispersive x-ray microanalysis (TEM–XEDS), and gene expression in order to investigate the effects of these heavy metals on cell growth, cellular structure, bioaccumulation, and cellular distribution. In addition, we aim to understand the genetic basis of resistance/tolerance to these metal(loid)s. Unlike other microalgae, the *C. acidophila* genome is not completely sequenced, and therefore, research on candidate genes like the phytochelatin synthase *CaPCS2* and its role in arsenic and cadmium resistance is much needed. This gene has been obtained in *C. acidophila* by horizontal transfer from bacteria and is not found in other microalgae [19]. Its function in *C. acidophila* could be related to cadmium and arsenic chelation and accumulation and therefore contribute to the resistance against metal(loid)s.

## 2. Materials and Methods

### 2.1. Algae Isolation and Cultivation

*Chlamydomonas acidophila* RT46 was isolated from Rio Tinto (Huelva, Spain) [20]. The microalgae were grown for 7 days in BG11 medium (pH 2) [29], supplemented with an f/2 mix vitamin solution [30]. Cell cultures were maintained at 20 ± 1 °C under white light illumination fluorescents tubes (70 µEs^−1^ m^−2^ irradiance) with 16:8 LD cycles.

### 2.2. Cytotoxicity Assays. Evaluation of ROS Generation

Cultures in exponential growth phase were exposed to a series of increasing concentrations of each toxic metal(l)oid: 0.1–10 µM Cd (CdCl_2_ · 2 ½ H_2_O, 99.99%, Sigma-Aldrich), 0.1–20 mM As(III) (NaAsO_2_, Sigma-Aldrich) or 1–50 mM As(V) (Na_2_HAsO_4_ · 7H_2_O, Sigma-Aldrich). All treatments were maintained for 24 h. Membrane integrity, an indirect sign of cellular viability, was assessed by propidium iodide (PI) fluorochrome (2.5 µg/mL). Fluorescent cells were detected by using the flow cytometer FACScaliburg (Becton-Dickinson) equipped with Cell Quest software. Red fluorescence due to cells with damaged or disrupted cell membranes (PI^+^) was collected by the Fl3 channel (670 nm LP). At least three replicates of each experiment were made. The Probality Unit model [31] and the statistical Statgraphics Centurion XVI program (Statgraphics Technologies Inc., Fauquier, VA, USA) (confidence 95%, *p* < 0.05) were applied to calculate metal concentration that is lethal to 50% of the population (LC_50_).

To detect reactive oxygen species (ROS) formation in control (untreated) and treated populations, we used the hydroethidine (HE) fluorochrome (Sigma-Aldrich). The fluorogenic probe is widely used for detecting intracellular superoxide. Stock solution was prepared in dimethylsulfoxide (DMSO) at a final concentration of 3 mM. Inside the cell, HE is selectively oxidized by superoxides to form 2-hydroxyethidium (2-E^+^OH), a fluorescent compound (λ excitation = 500–530 nm, λ emission = 590–620 nm) [32]. We have detected that both fluorochromes (PI and HE) cannot be used in acidic conditions. For that reason, cell cultures were washed in 0.01 M HCl-Tris buffer, pH 6.8, and immediately, the dye was added. Our earlier testing showed that this procedure does not produce any effect on cell viability.

### 2.3. Ultrastructural analysis by Transmission Electron Microscopy (TEM)

To observe the potential ultrastructural alterations, controls (untreated cells) and cell populations exposed to sublethal treatments of metal(l)oids during 24 h to 0.5 µM Cd, 5 mM As(III) or 25 mM As(V) was analyzed by TEM using previously reported protocols [33,34]. Ultrathin sections were stained with uranyl acetate and lead citrate and, finally, mounted on copper grids and examined with a Jeol JEM 1010 transmission electron microscope operating at 75 kV.

### 2.4. TEM–Energy Dispersive X-Ray Microanalysis (XEDS)

To assess the metallic nature of electron-dense deposits observed inside the cells by TEM, cell samples exposed to the same metal treatments were processed for TEM–energy dispersive X-ray microanalysis (XEDS). XEDS makes use of the X-ray spectrum emitted by a solid sample bombarded with a focused beam of electrons to obtain a localized chemical analysis. In this case, cells were not post-fixed with osmium tetroxide and thin sections were 1 µm in width. Double contrasting with uranyl acetate and lead citrate was not applied. Cu girds were used to eliminate the interference with the As or Cd emission spectra. Microanalysis was made with a transmission electron microscope Jeol JEM 2100 (Acceleration voltage 200 kV, Resolution limit 0.25 nm), equipped with an XEDS microanalysis system (Oxford Inca) and operating at 100 kV.

### 2.5. Isolation of Total RNA. Primer Design and Quantitative Real-Time RT–PCR (qRT–PCR)

Cell cultures in stationary phase were exposed to final concentrations of Cd 1 µM, As(III) 1 mM or As(V) 5 mM. These treatments were maintained during 1, 3, and 24 h. Cells were collected by centrifugation for 5 min at 1398× *g*. Total RNA was isolated with TRI Reagent Solution (Ambion, Life Technologies, CA, USA) following the manufacturer’s protocol. Cells were disrupted using a FastPrep bead beating instrument (Bio 101). To remove possible DNA contamination, all samples were treated with DNase I (RNase free) (Ambion). RNA integrity was verified using a 2100 Bioanalyzer (Agilent Technologies, Santa Clara, CA, USA). Good quality RNA with preserved 18 and 28 S rRNA bands was used for real-time polymerase chain reaction (RT–PCR) analysis.

We analyzed by quantitative RT–PCR the expression level of the previously reported phytochelatin synthase gene *CaPCS2* from *C. acidophila* [19]. Details of primer design and qRT–PCR procedure are consigned in Table 1. The standard line parameters (amplification efficiency, slope, and correlation coefficient) are reported in Table 2. Analysis of relative gene expression was carried out according to the standard-curve quantification method [35] from, at least, four independent experiments (each performed in duplicates).

cDNA samples were amplified in duplicate in 96-well microtiter plates. Each qPCR reaction (20 µL) contained 10 µL of SYBR Green (Takara), 0.4 µL of ROX as passive reference dye (Takara), 1 µL of each primer (at 40 nM final concentration), 3.6 µL of ultrapure sterile water (Roche), and 4 µL of a 10^−1^ dilution of cDNA. PCR primers (Table 1) were designed using the “Primer Quest and Probe Design” online-application from IDT (Integrated DNA Technologies). Both *18 rRNA* and *β-actin* were used as endogenous control or normalizer genes. Melting curves were obtained and primers specificity was tested by confirming each PCR product by gel electrophoresis and sequencing. Real-time PCR reactions were carried out in an iQ5 real-time PCR apparatus (Bio-Rad, Laboratories, Inc., Madrid, Spain) and the thermal cycling protocol was as follows: 5 min at 95 °C, 40 cycles (30 s at 95 ° C, 30 s at 55 °C and 20 s at 72 °C), 1 min at 95 °C, and 1 min at 55 °C. All controls (no template controls (NTC) and Retro-transcription (RT) negative controls) were negative. The standard line parameters (amplification efficiency, slope, and correlation coefficient) are reported in Table 2. Amplification efficiency (E) was measured by using 10-fold serial dilutions of a positive control PCR template.

### 2.6. Statistics

Statistical analysis for quantitative RT–PCR was carried out using the pair wise fixed reallocation randomisation test (REST-MCS β version 2 software, Munich, Germany) [36]. One-way analysis of variance test (ANOVA) was applied to assess the significance of differences between control (non-treated) and treated cells. The results are expressed as means ± standard error and statistical significance is defined as *p*-value < 0.001.

## 3. Results

### 3.1. Cell Viability and ROS Generation Assessments

Cd is the most toxic heavy metal for this acidophilic strain. In fact, the LC_50_ for Cd was as low as 1.94 µM. However, *C. acidophila* showed high resistance to arsenic, a non-essential metalloid. The toxicological response was different depending on the chemical form of arsenic used in this bioassay. Arsenite (As(III)) is remarkably more toxic than arsenate (As(V)) to this photosynthetic microorganism. The LC_50_ values were 10.91 mM for As(III) and 41.63 mM for As(V).

**Figure 1:**
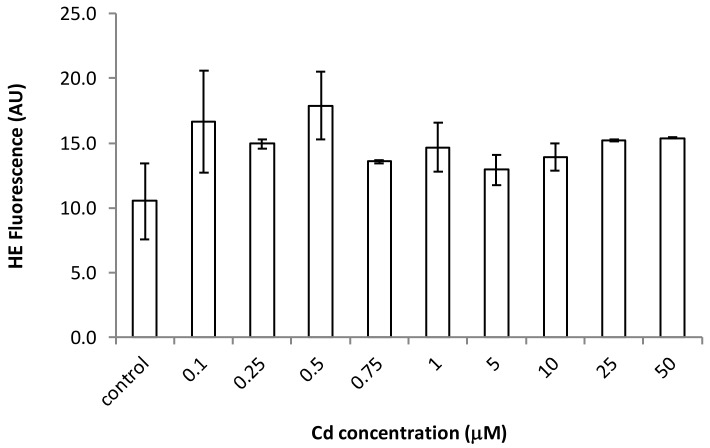
Increase of hidroethidine fluorescence in cultures exposed to different Cd treatments. The analysis fluorescence results indicated that there is not a statistically significant difference (*p* > 0.05) between cadmium treatments and non-treated cells (control). AU—arbitrary units.

Since some heavy metals and metalloids are potent oxidants, we evaluated the ROS generation, mainly of superoxide anions, using the fluorochrome hydroethidine. Our results indicated that there is not a statistically significant difference (*p* > 0.05) between the cadmium exposed populations and non-treated cells (control populations), at least not under the Cd concentrations used (0.1–50 µM; Figure 1).

**Figure 2:**
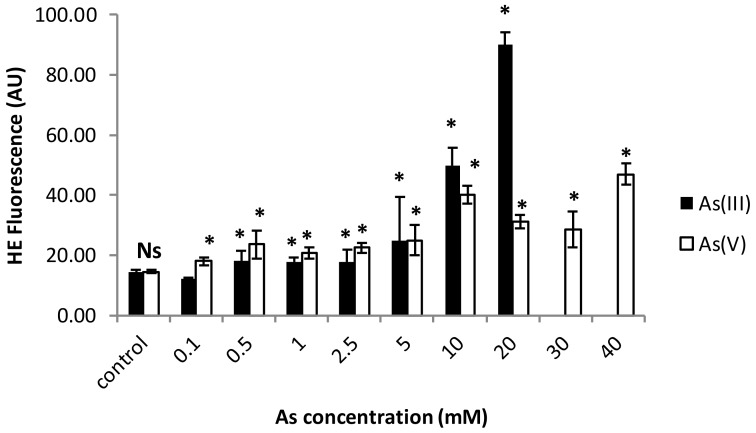
Increase of hidroethidine fluorescence in cultures exposed to different arsenite (As(III)) or arsenate (As(V)) treatments. * (asterisks) indicate significant differences from control (*p* < 0.001). Ns— non-significant.

Again, for arsenic, data of superoxide generation levels presented significant differences depending on the two inorganic forms of arsenic used (Figure 2). Under As(III), the most toxic form for this strain, a statistically significant difference between treated and control populations was observed, but the increase in the superoxide anion generation is clearer under the highest concentrations (10 and 20 mM As(III); Figure 2). In this case, there was a directly proportional relationship between the superoxide increment and the As(III) concentration. ROS generation was significantly lower for As(V) treatments, although a statistically significant increase was assessed under all applied concentrations (0.1–40 mM As(V)).

### 3.2. Ultrastructural Alterations under Cadmium and Arsenic Treatments

**Figure 3:**
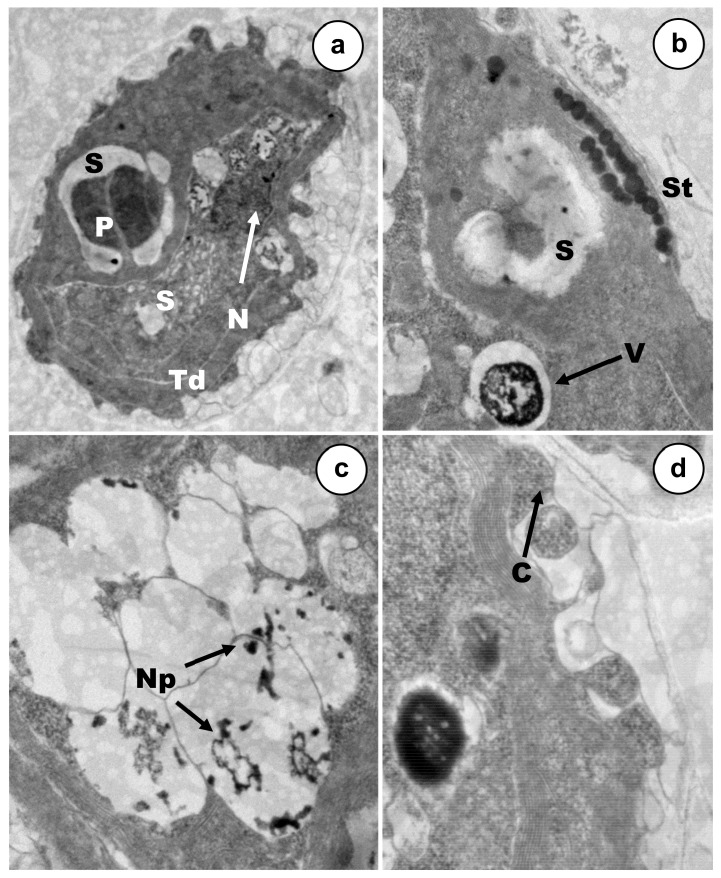
TEM micrographs of cells exposed to Cd 0.5 µM. (**a**) General appearance of a treated cell (15k ×). (**b**) Detail of pyrenoid, stigma, and cell vacuole (50k ×). (**c**) Nanoparticles-like inside vacuoles (60k ×). (**d**) Discharge of exopolymers on cellular surface (60k ×). NP—Nanoparticles-like; S—starch deposits; P—pyrenoid; St—stigma; V—vacuole; Td—thylakoids; C—capsule.

**Figure 4:**
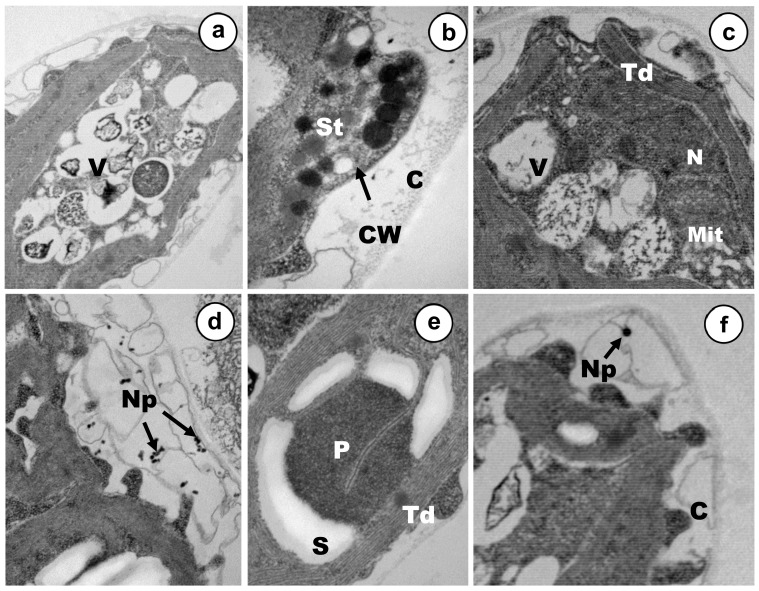
Ultrastructure of arsenate treated vegetative cells. (**a**) Intense vacuolization and bioaccumulation, (15k ×). (**b**) Stigma degeneration, (50k ×). (**c**) Details of the particulate vacuolar content, capsule growth and nuclear alteration, (25k ×). (**d**) Nanoparticle-like formation inside cytoplasmic vacuoles, (50k ×). (**e**) Strong starch accumulation around the pyrenoid, (50k ×). (**f**) Nanoparticle-like entrapped in the capsule, (25k ×). S—Starch deposits; P—pyrenoid; St—stigma; V—vacuole; Td—thylakoids; C—capsule; Np—nanoparticles-like (Np); Mit—mitochondria; CW—cell wall; N—nucleus.

**Figure 5:**
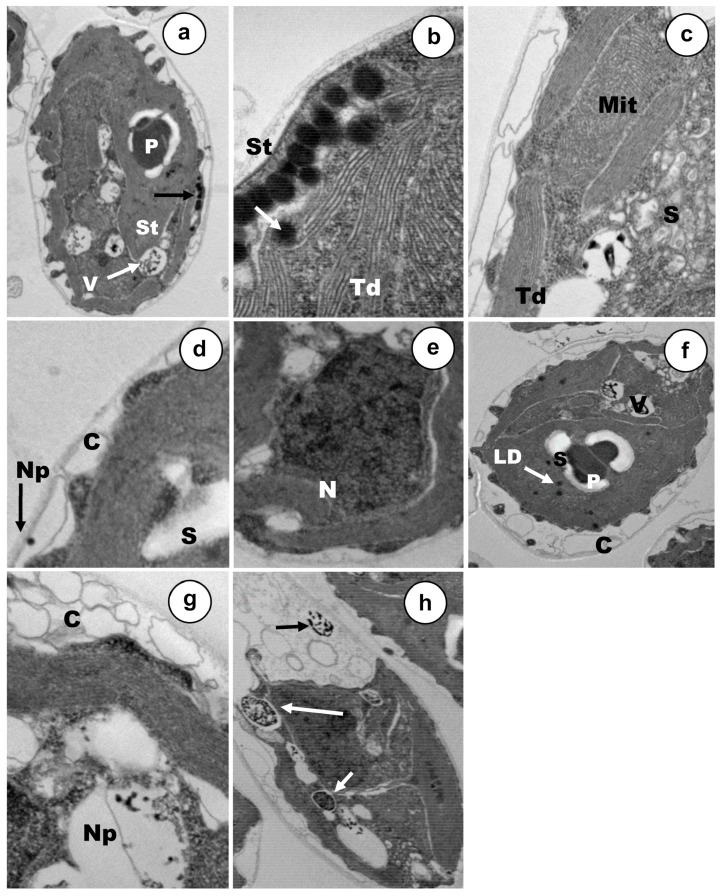
TEM micrographs of arsenite treated cells. (**a**) View of a vegetative cell in longitudinal section; (12k ×), (**b**) Severe disorganization of photoreceptive stigma (60k ×). (**c**) Starch deposits, mitochondria, and alterations in thylakoid membranes (40k ×), (**d**) Nanoparticles-like embedded in the polysaccharidic capsule, (60k ×). (**e**) Altered nuclear pattern (60k ×). (**f**) Vegetative cell showing lipid droplets, bioaccumulation, starch deposits, and exopolymer discharge (12k ×). (**g**) Cytoplasmic nanoparticles-like into vacuoles (40k ×). (**h**) Full vacuoles expelled outside cell (12k ×). S—Starch deposits; P—pyrenoid; St—stigma; V—vacuole; Td—thylakoids; C—capsule; Np—nanoparticles-like; Mit—mitochondria; CW—cell wall; N—nucleus.

**Figure 6:**
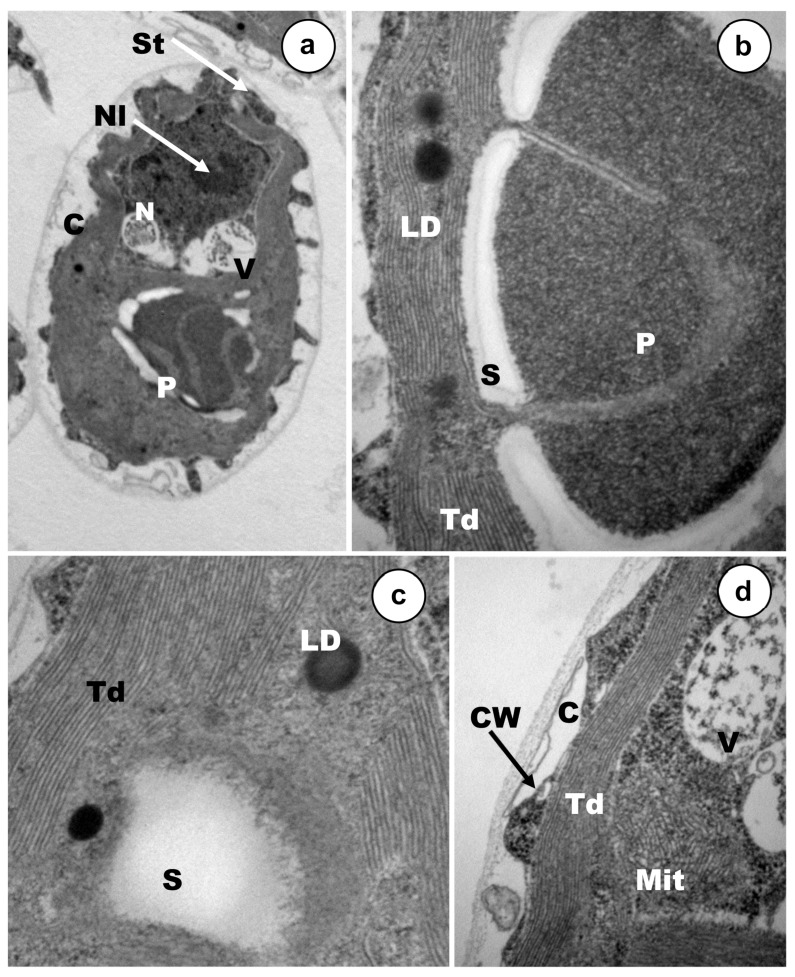
Ultrastructural features of *C. acidophila* RT46 control cells. (**a**) Longitudinal section of a vegetative cell (15k ×), (**b**) cytoplasm view in detail, showing the thylakoidal membranes, lipid droplets, pyrenoid, and the starch deposits around this structure (80k ×), (**c**) thylakoid arrangement and energy reserves (80k ×), (**d**) cellular surface appearance and a mitochondrial ultrastructure (50k ×). S— Starch deposits; P—pyrenoid; St—stigma; V—vacuole; Td—thylakoids; C—capsule; Np—nanoparticles-like; Mit—mitochondria; CW—cell wall; N—nucleus; Nl—nucleolus.

Cell populations were exposed to rather low concentrations (12–18% mortality), of each metal(oid), Cd, As(III) or As(V) during 24 h (Figure 3, Figure 4 and Figure 5). The main ultrastructural details of control cells are shown in Figure 6. *C. acidophila* RT46 was very sensitive to Cd. This is consistent with the severe ultrastructural alterations that were observed after its exposure to a concentration of 0.5 µM of this heavy metal. The most remarkable sign of chemical stress in cells was a drastic increment in starch reserves. This sugar bioaccumulation increased in deposits around pyrenoid (Figure 3a,b). Besides, new starch grains appeared in certain regions of the cytoplasm (Figure 3a). Under this Cd concentration, no ultrastructural changes in the stigma were observed (Figure 3b). However, it was possible to detect an intense process of cytoplasmic vacuolization in many cells. Inside these vacuoles, there were tiny electron-dense particles, which might be metal particles (nanoparticles). When each vesicle became full, it was expelled out of the cell (Figure 3b,c). In response to Cd, a strong exopolysaccharide discharge took place, increasing the width of the capsule (Figure 3d). Finally, the chromatin distribution pattern in the nucleus underwent changes and the nucleolus became non-visible (Figure 3a). 

Ultrastructural modifications presented specific traits and main targets depending on the arsenic oxidation state used in exposures, at least under the tested concentrations. Stigma were severely affected by the presence of As(V), but mitochondria remained apparently unaffected (Figure 4b). The thylakoids of the sole chloroplast underwent some disorganization in some cells, whereas in other ones, the ultrastructural organization of this organelle seemed to be rather well and it was possible to observe the characteristic disc stacking (Figure 4a,c,e). A strong increment of starch reserves was detected in the cytoplasm, being higher in those deposits placed around the pyrenoid (Figure 4d,e). In response to arsenate stress, two main changes were assessed in *C. acidophila*, which are probably involved in cellular resistance. Firstly, many vacuoles that have been filled progressively, containing electron-dense micro/nano-particles, could be noted in most of the cells (Figure 4a,c). Secondly, a large number of exopolymers were expelled on the cell surface and many discrete As(V) nanoparticles could be clearly observed entrapped inside the growing capsule (Figure 4d,f). With regard to the nucleus, the central nucleolus could not be detected in treated cells and several chromatinic masses appeared (Figure 4c).

Arsenite (Figure 5) caused severe ultrastructural alterations in the photosynthetic elements of *C. acidophila*. Stigma and thylakoids displayed intensive disorganization in almost all the cells (Figure 5a–c). Mitochondria were also damaged (Figure 5c) and it was possible to detect an increase in both the lipid droplets content (in some cells) and starch depositions (in all cells) (Figure 5a,c,f). On the contrary, Golgi apparatus ultrastructure remained apparently unaffected (Figure 5b). Nuclear appearance underwent some changes and the nucleolus became invisible (Figure 5e). Both bioaccumulation and biosorption might be involved in As(III) resistance in this *Chlamydomonas* strain. Electron-dense discrete nanoelements appeared embedded inside a thick polysaccharidic capsule (Figure 5d,f). Furthermore, there was a gradual nanoparticle accumulation into a series of cytoplasmic vacuoles (Figure 5g). When each vacuole became full, this organelle moved towards the capsule where the metalloid content was discharged (Figure 5h).

### 3.3. TEM Energy Dispersive X-Ray Microanalysis (XEDS)

**Figure 7:**
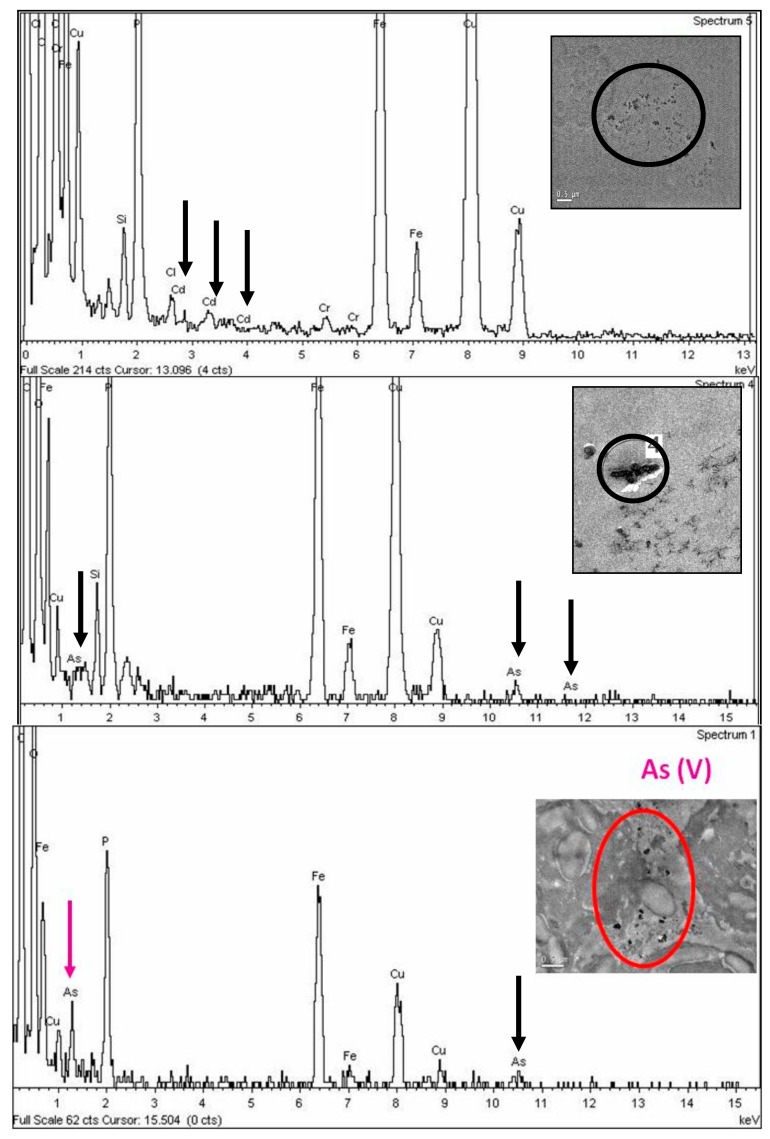
Spectra of *C. acidophila* RT46 after metal(l)oids exposures (**a**) Cd, (**b**) As(III), (**c**) As(V).

TEM energy dispersive X-ray microanalysis (TEM–XEDS) was extensively utilized to analyze the elemental composition of intracellular electron-dense deposits formed by *C. acidophila* after metallic treatments (Figure 7). Analysis of the energy-dispersion spectra of exposed populations indicated that cells of *C. acidophila* have been able to bioaccumulate all the metal(l)oid forms used in experimental treatments (Figure 7a–c). Curiously, intracellular levels of phosphorous detected were also very high. All spectra showed prominent peaks of Fe and Cu, which respectively corresponded to the accumulation of Fe in this strain and to the Cu nature of the used girds. 

### 3.4. Analysis of CaPCS2 Gene Expression by Quantitative RT-PCR

Table 3 illustrates the relative fold induction values for the *CaPCS2* gene, a phytochelatin synthase gene characterized by Olsson et al. [19] under Cd treatments. In this study, we have included two new stress treatments (As(III) and As(V)), applied during three time periods: 1 h (short exposure), 3 h (medium exposure), and 24 h (long exposure). Results showed a differential gene expression depending on both the chemical nature of the toxic agent and the treatment length. In general, the highest induction for *CaPCS2* mRNA expression levels was obtained after 3 h exposures in all the cases. After this time of As(V) treatment, the *CaPCS2* gene was induced to the highest level obtained, about 2900-fold. Under the same exposition time with As(III) or Cd, induction levels of 573- and 1275-fold on average, respectively, were observed (Table 1). Lower but significant overexpression levels were reached still after 24 h treatments, yielding in this case relative fold-induction values ranking from As(V) > As(III) > Cd. Noteworthy, the induction data from As treatments were remarkably different, depending on the oxidation form used in bioassays. So, As(V) exposure caused higher overexpression of *CaPCS2* gene (402-fold on average) than As(III) (63-fold on average), despite the fact that this oxidation state was less toxic for *C. acidophila.* Similar differences were detected under short exposures (1 h). Under these conditions, As(V) produced again higher gene expression induction levels (585-fold on average) than As(III) (around 14-fold).

## 4. Discussion

### 4.1. Cytotoxicity and ROS Induction

Microalgae are unicellular microorganisms, which constitute a key trophic level in aquatic ecosystems because they are responsible for a large proportion of total primary production [37]. Data from *C. acidophila* RT46 showed that Cd tolerance was not as high as we had assumed previously, considering the Cd concentration (4.1 mg/L) of the sampling point at the river [26]. The LC_50_ calculated after 24 h exposure to this metal was quite low: 1.94 µM (0.217 mg/L). After a review of published reports, we found that certain species have been reported as more tolerant of this metal, i.e., *Chlamydomonas moewusi* [38], *Pediastrum simplex* and *Synedra acus* [39], *Chlorella* sp. [40], and *Isochrysis galbana* [41]. Then again, other species seem to be more sensitive to Cd, for instance, *Scenedesmus obliquus* (EC_50_ = 0.058 mg/L) [42], *Nitzschia palea* (0.0276 mg/L) [43], and *Pseudokirchneriella subcapitata* (0.6 µM) [44]. *C. acidophila* has also shown an intraspecific variation in Cd tolerance. A strain of *Chlamydomonas* isolated from Rio Tinto and tested more than a decade ago presented an average of 50% growth inhibition at 0.2 mM Cd [45]. A significantly lower EC_50_ (14.4 µM) was reported for a strain from an acid lake [46].

Surprisingly, the Cd LC_50_ value shown by our strain RT46 was around 19-fold lower than the Cd concentration (4.1 mg/L) measured at the same time during the sampling point of the river. We assume this remarkable decrease in Cd tolerance of our strain to be caused by the long time it has been grown in the laboratory under appropriate conditions (e.g., nutrients, pH) but without metals. Several hypothetical reasons can explain these apparently conflicting results: Firstly, it is known that metal interactions cause severe changes in biotoxicity. Consequently, the high levels of Cd, Cr, As, Ni, Zn, Cu, and Fe usually presented in Rio Tinto might cause severe interferences on cellular toxicokinetics and toxicodynamics for each individual metal. Alternatively, it has been shown that some eukaryotic microorganisms can acquire or increase the level of metal stress tolerance and even develop novel metabolic abilities when they are exposed to selective pressure. In yeast and ciliates, the increment of the tolerance to these metals in their environment was attributed to the specific metallothionein gene amplification [47,48]. Experimental evidence supports the idea that environmental heterogeneity contributes to maintaining genetic variation in fitness [49]. The observed Cd toxicity differences between bioassays and environmental data in *C. acidophila* attract even more attention considering the phosphate concentrations in both media: BG11 medium has a high phosphate concentration (estimated as 0.22 mg/L), whereas in Río Tinto the phosphate concentration was below the limit of detection of the used methodology [50]. A recent report from the acidic lakes of Germany indicated that at high local iron concentrations (260 mg total Fe/L; i.e., 15 mg free ionic Fe^3+^/L), reduced Pi-incorporation by 50% and resulted in Pi-limited photosynthesis [51]. Therefore, it might be that Pi-incorporation decreased effectively and led to Pi-limitation in the acid Fe-rich Tinto river. Experimental evidence pointed out that poly-P are not the sole mechanism involved in Cd tolerance in microalgae found in acid Fe-rich environments.

Studies about the cytotoxicity of arsenite and arsenate are very scarce [5,52]. Variation and contradictions in the level of As tolerance reported in the literature makes estimating the most toxic arsenic species for each microorganism unreliable. In our strain of *C. acidophila* RT46, the LC_50_ values obtained were 10.91 mM (817.7 mg/L) for As(III) and 41.63 mM (3119 mg/L) for As(V). So, we conclude that As(III) is almost 4-fold more toxic than As(V). Moreover, this is the most As tolerant microalga (considering both As(III) and As(V)) reported until present. In our strain, arsenic tolerance levels were much greater to those reported for other microalgae, such as *Monoraphidium arcuatum* (IC_50_As(III) = 15 mg/L, IC_50_As(V) = 0.25 mg/L) [53], *Chlorella* sp. CE-35 (IC_50_As(III) = 27 mg/L, IC_50_ As(V) = 1.15 mg/L) [54], *Scenedesmus obliquus* (IC_50_As(V) = 64.86 µg/L) and *Chlamydomonas reinhardtii* (IC_50_As(V) = 33.50 µg/L) [55], and even in comparison with other acidophilic microalgae [56]. Equal biotoxicity for As(III) and As(V) was evaluated for *Chlorella* sp. (25 mg/L) [57] and *Chlorella salina* [58]. Several physicochemical factors (pH, phosphate, certain metals) seem to be involved in the modulation of uptake and biosorption of As and, therefore, in the biotoxicity [58,59,60,61].

Cadmium is a transition metal that frequently appears as a pollutant of air, soil, and waters. It is very toxic for microorganisms, animals, plants, and humans. Oxidative stress is one of the main mechanisms attributed to Cd toxicity [23,62]. However, mechanisms of oxidative stress generation by this metal have not been yet fully understood in photosynthetic organisms [57,63]. Although Cd cytotoxicity bioassays using microalgae are quite numerous, only two direct reports of ROS induction by Cd have been published. Using dihydrorhodamine 123, a significant increase in H_2_O_2_ production was detected in *C. reinhardtii* [63]. In *Euglena gracilis*, Cd exposure induced an increment of ROS generation that was observed by the application of the less specific fluorophore dihydrofluorescein diacetate [64,65]. In the current study on *C. acidophila*, the comparison between the generation of superoxide and the Cd concentrations showed that levels of this anion did not rise in a statistically significant way, so it is possible that Cd generates other types of ROS. A marked increase of fluorescence was detected after arsenite and arsenate exposures; thus both oxidation states induced the generation of superoxide in *C. acidophila.* In animals, many studies have indicated that oxidative stress is involved in arsenic cyto- and genotoxicity [66]. In microalgae, As(V) exposure induced moderate ROS production, while arsenite (As(III)) resulted in no detectable ROS signal in *C. reinhardtii* [67]. Indirect evidence of free radical generation after arsenic exposure was substantiated by alterations in the functioning of the antioxidant enzymes and confirmed by proteomic analysis, where protein synthesis involved in ROS scavenging and defense were detected [68]. Moreover, the biosynthesis of phytochelatins and other thiol-rich compounds have been also detected to act as strong antioxidant molecules [27,59].

### 4.2. Ultrastructural Changes

Studies of ultrastructural alterations due to metal(loid)s exposures provide useful information about the main cell targets for metals and resistance mechanisms. Treatments with Cd, As(III), and As(V) caused in *C. acidophila* several common modifications associated with environmental chemical stress, although the change pattern was specific for each metal treatment. An intense vacuolization was the most evident ultrastructural modification in the cytoplasm after metal exposures. Intense vacuolization due to Cd uptake has also been reported in *Skeletonema costatum* [69], *Chlamydomonas reinhardtii*, and other strains of *C. acidophila* [70,71]. With regard to arsenic exposure, vacuolization has been reported in *Skeletonema quadricaudata* in presence of arsenite [72]. Another remarkable change was a drastic increment of starch reserves; these accumulations appeared distributed randomly in the cytoplasm, especially around the pyrenoid. An increase in carbohydrate reserves, as a consequence of metal or other stresses, has been reported in other microalgae [45,73,74]. Unlike in other species, we have only observed an increment in lipid reserves after As(III) exposure. Lipid and starch are key energy reserves for microalgae and their biosynthesis seems to involve the same C3 precursor pool. They serve as electron sinks under conditions where photosynthesis or metabolism of an exogenous carbon source is still active but growth is limited by environmental stresses [75]. In our opinion, all of these alterations (vacuolization, starch deposits, lipid droplets, and metal bioaccumulation) might explain the increase in both cellular size and granularity, as had been previously detected by flow cytometry after Cu and Cd treatments in protists [45,76]. Alternatively, other authors suggested that the cell size increase was due to a decrease in cell division rate by metallic stress [77].

No previous studies have been done in microalgae to analyze ultrastructural modifications and cytotoxic effects caused by As(III) or As(V) by flow cytometry. The treatments used for As(III) and As(V) in *C. acidophila* caused severe disorganization in stigma and thylakoids, but no ultrastructural modification of these organella was detected after Cd exposure. As in C. *reinhardtii*, the eyespot apparatus is usually composed of two highly ordered layers of carotenoid-rich lipid globules, which has a very electron-dense appearance. This photoreceptive apparatus is present in most of flagellated photosynthetic protists. Previously, it has been suggested that Cr produced severe ultrastructural alterations and decomposition of the overall absorption spectra in pigment constituents of both structures in *C. reinhardtii* and other species [78,79]. Degradation of stigma under metal exposure has been reported in microalgae previously, although without the publication of micrographs or other evidence [73,74]. Mitochondrial ultrastructure was irreversibly damaged by As(III), due to the high toxicity of this cation for *C. acidophila*, similar to other organisms [24,80]. All treatments caused alterations in the nuclear pattern of *C. acidophila*, supporting the genotoxicity attributed to Cd, As(III) and As(V) in unicellular organisms, plants, and animals [80].

### 4.3. Analysis of Mechanisms Involved in Tolerance

Bioaccumulation and biosorption are two main resistance mechanisms against metal(loid)s used by microalgae and other biological systems, although there are also other well-characterized protection mechanisms [52,81]. Our micrographs indicate that both means of cation immobilization have been involved in the resistance to Cd and As. Furthermore, it is very probable that the export metal mechanism (mediated by ATPases) will also be involved in the response to these metal/metalloids. Nanoparticles/particle-like formations develop inside cytoplasmic vacuoles. When the content of these vacuoles became full, they are discharged outside the cells. Due to the existence of several mechanisms involved in the response of this strain to the solution, it is impossible to localize (by this methodology) metal accumulates in determinate subcellular cytoplasmic sites (e.g., mitochondria, endoplasmic reticulum, Golgi, peroxisomes). Additionally, a massive discharge of exopolymers on the cell surface lead to an increase in the thickness of the capsule, where metallic deposits are entrapped. The chemical nature of these deposits was verified by TEM–XEDS. In some microalgae, data indicate that Cd bioaccumulation might be mainly mediated by the tripeptide GSH and its polymeric derivatives, known as phytochelatins [81,82]. There is little experimental evidence of As bioaccumulation in microalgae mediated by phytochelatins [56,59,83], although in higher plants, the participation of these peptides in As accumulation has been corroborated [84].

For the first time in microalgae, we have provided direct evidence of the involvement of a phytochelatin synthase gene in arsenic resistance However, phytochelatins are not the only mechanism involved in resistance against the biotoxicity of these metalloid forms. The high levels of phosphorus detected by TEM–EDX inside cells also support the role of polyphosphate in arsenic resistance. This phytochelatin synthase gene showed a very strong induction by cadmium [19]. In this study, we have shown by RT–qPCR that expression of *CaPCS2* was also much higher in the presence of As(III) and As(V), indicating that phytochelatins are involved in arsenic resistance in *C. acidophila*. Under arsenite/arsenate exposures, the strongest expression for this gene was obtained after 3 h of treatment. Therefore, phytochelatins might not represent the first barrier against the biotoxicity of these metalloid forms. The high levels of phosphorus detected inside cells in all treatments (see TEM–EDX spectra) also support the role of polyphosphate in metal resistance [27,52]. Moreover, in recent studies by X-ray absorption near edge structure (XANES) spectroscopy in several species of marine microalgae, Cu resistance and bioaccumulation has been associated with poly-phosphate [85]. Glutathione and phytochelatins contain free thiolic groups that can reduce As(V) to As(III) and they can also immobilize both cationic forms. Furthermore, the capacity to reduce As(V) to As(III) as the first stage in a detoxification process has been suggested for some microalgae [53,54,60]. Redox transformations between both inorganic forms of As are quite usual in prokaryotes [86]. However, they have not been well documented in microalgae, although in the *C. reinhardtii* genome, two arsenate reductase genes were identified, cloned, and expressed on an *Escherichia coli* strain [87].

## 5. Conclusions

*Chlamydomonas acidophila* RT46 was quite sensitive to Cd but very tolerant to As, although arsenite was almost four-fold more toxic than arsenate. Both As inorganic species induced the generation of remarkable superoxide anion levels in cellular populations. Intense vacuolization and a drastic increase of starch and lipid reserves were detected after Cd and As treatments. Moreover, As(III) and As(V) caused severe disorganization in the eyespot or stigma and thylakoids, but no ultrastructural modification of these organella was detected after Cd exposure. Metal(loid) bioaccumulation and biosorption might be implicated in the tolerance of *C. acidophila* to Cd and As. TEM–XEDS analysis and qRT–PCR data suggested that poly-phosphate and phytochelatins are involved in bioaccumulation. Additionally, a massive discharge of exopolymers on the cell surface occurred in the presence of the toxics, producing an increase in the thickness of the capsule, where metallic deposits were entrapped.

## Figures and Tables

**Table 1:** Primer name for target gene and the expression control genes (housekeeping), and forward or reverse sequence are indicated. The optimal annealing temperature was 55 °C in all cases. *CaPCS2* = phytochelatin synthase gene. All oligonucleotide primers used in the study were previously reported [19].

PCR Primer	Gene	Sequence (5′ to 3′)	F/R
CaPCH2_3F	*CaPCS2* (target gene)	TGGGATTGGGATATTGTGCT	F
CaPCH2_2R		ATCTGTTTATGCCCCTGCAC	R
18SrRNAF	*18S rRNA* (housekeeping)	TCAACTTTCGATGGTAGGATAGTG	F
18SrRNAR		CCGTGTCAGGATTGGGTAATTT	R
RT46_ACT1	*Actin* (housekeeping)	CTCACTCTCAACATTCCAGCAA	F
RT46_ACT2		GGGCCCGCTCTCATCATACTC	R

PCR—polymerase chain reaction; F—forward sequence; R—reverse sequence.

**Table 2:** Quantitative real-time polymerase chain reaction (RT–PCR) standard-curve parameters for target gene (*CaPCS2*) and the expression control (housekeeping) genes.

Gene	E	S	R^2^
***CaPCS2***	1.95	−3.43	0.99
**18S rRNA**	2.09	−3.12	0.98
**Actin**	2.10	−3.2	0.98

E—amplification efficiency; S—slope, R^2^—correlation coefficient.

**Table 3:** Fold induction values and standard deviation (SD) from the qRT–PCR analysis of *Chlamydomonas acidophila CaPCS2* gene obtained by quantitative RT–PCR after As or Cd exposure during 1, 3, and 24 h. The results are expressed as relative mRNA expression normalized to both control genes (housekeeping). Data are means ± SD of three independent experiments. Significant differences between treated and non-treated cells are indicated by *p* < 0.001.

Treatment	Exposure (h)	Fold Induction (±SD)	Author
**As(III)**	1	14 ± 5	This study
	3	526 ± 317	
	24	63 ± 20.4	
**As(V)**	1	585.4 ± 239	This study
	3	2959 ± 649	
	24	402 ± 116	
**Cd**	1	2.0 ± 1.1	Olsson et al. [19]
	3	1275.3 ± 218.8	
	24	47.8 ± 24.1	
